# Malformed vertebrae: a clinical and imaging review

**DOI:** 10.1007/s13244-018-0598-1

**Published:** 2018-04-03

**Authors:** Apeksha Chaturvedi, Nina B. Klionsky, Umakanthan Nadarajah, Abhishek Chaturvedi, Steven P. Meyers

**Affiliations:** 1 Department of Pediatric Radiology, Golisano Children’s Hospital, University of Rochester Medical Center, 601, Elmwood Avenue, Rochester, NY 14642 USA; 2 Sri Jayewardenepura General Hospital, Colombo, Sri Lanka

**Keywords:** Vertebral malformations, Children, Spine, Magnetic resonance imaging, Developmental

## Abstract

A variety of structural developmental anomalies affect the vertebral column. Malformed vertebrae can arise secondary to errors of vertebral formation, fusion and/or segmentation and developmental variation. Malformations can be simple with little or no clinical consequence, or complex with serious structural and neurologic implications. These anomalies can occasionally mimic acute trauma (bipartite atlas versus Jefferson fracture, butterfly vertebra versus burst fracture), or predispose the affected individual to myelopathy. Accurate imaging interpretation of vertebral malformations requires knowledge of ageappropriate normal, variant and abnormal vertebral morphology and the clinical implications of each entity. This knowledge will improve diagnostic confidence in acute situations and confounding clinical scenarios.

This review article seeks to familiarize the reader with the embryology, normal and variant anatomy of the vertebral column and the imaging appearance and clinical impact of the spectrum of vertebral malformations arising as a consequence of disordered embryological development.

Teaching points

*• Some vertebral malformations predispose the affected individual to trauma or myelopathy.*

*• On imaging, malformed vertebrae can be indistinguishable from acute trauma.*

*• Abnormalities in spinal cord development may be associated and must be searched for.*

*• Accurate interpretation requires knowledge of normal, variant and abnormal vertebral morphology*.

**Electronic supplementary material:**

The online version of this article (10.1007/s13244-018-0598-1) contains supplementary material, which is available to authorized users.

The vertebral column and spinal cord develop early in gestation in a fine-tuned, sequential manner [1]. Any disruption of this normal sequence of events can lead to variations in the structural anatomy of the spine and spinal cord [2]. Structural malformations of the spine are often simple and may either go undetected or be discovered fortuitously. Occasionally, these may be complex with serious structural or neurological implications [2, 3]. These may occur sporadically, in isolation or as an accompaniment to multiorgan developmental malformations [4]. When symptomatic, these abnormalities can predispose the affected individual to biomechanical instability, spinal canal narrowing and myelopathy and can even be life-threatening. Developmental defects of cardiovascular, neurological, urinary and reproductive systems may be associated [5].

This article overviews the embryology of the vertebral column and imaging appearance and clinical impact of the spectrum of abnormalities arising as a consequence of disordered development. It is difficult to separate discussion of the spine from that of the enclosed spinal cord, so associated spinal cord/brain anomalies will be mentioned where applicable but will not be elaborated.

## Embryology

**Fig. 1:**
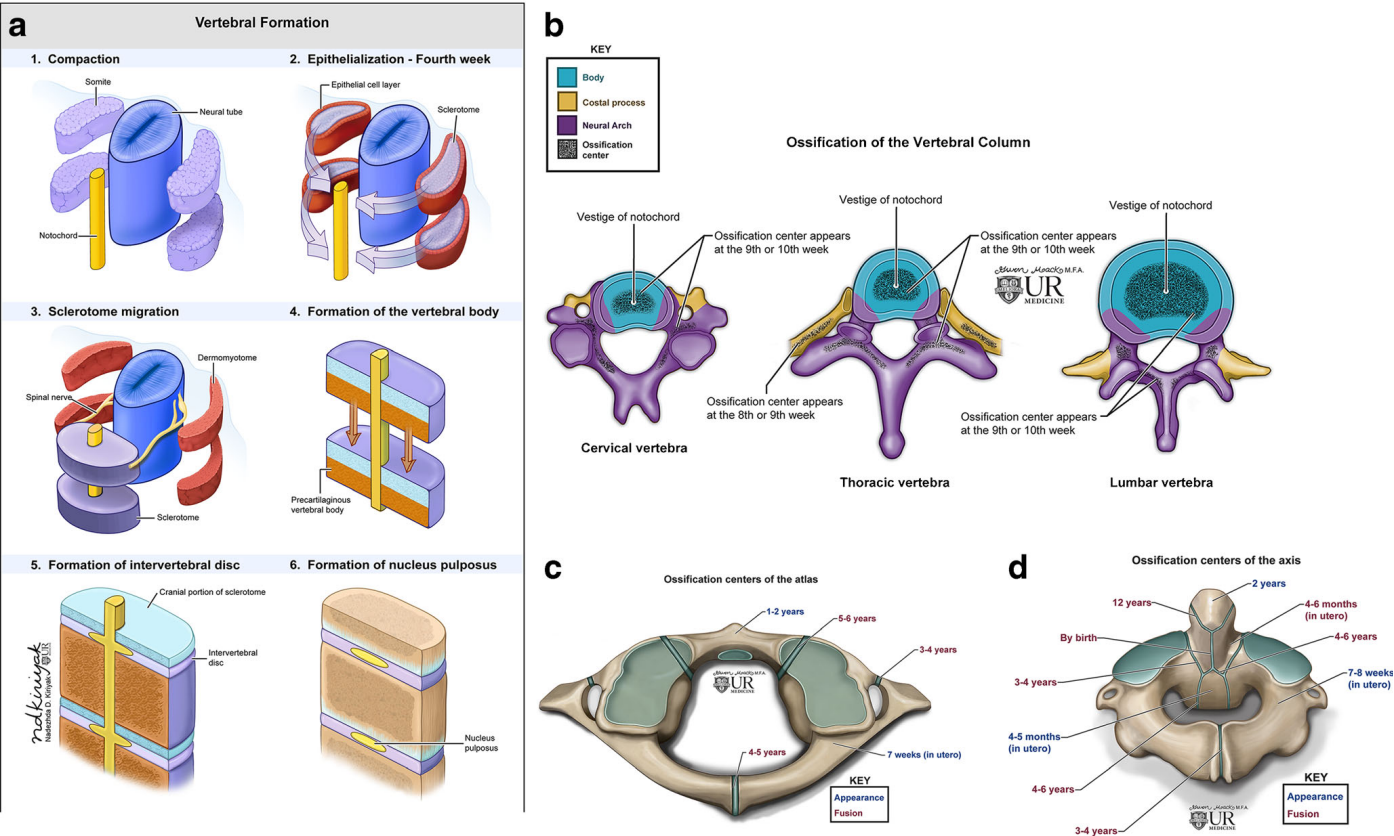
**a**-**d**: Embryology of vertebral bodies and intervertebral discs: Illustration (1–6) demonstrating the steps in development of vertebral bodies from sclerotomes. The sclerotomes migrate around the neural tube and the notochord forming the vertebral bodies, arches, transverse and spinous processes. Chondrification centres appear during the 6th week of development within a mesenchymal template, which enclose the notochord and developing neural tube. The notochord expands and develops into the nucleus pulposus of intervertebral disks. The six stages of vertebral development include: (1) gastrulation and formation of the somitic mesoderm and notochord, (2) condensation of the somitic mesoderm into somites, (3) formation of dermomyotomes and sclerotomes, (4) formation of membranous somites and re-segmentation with definitive vertebral formation, (5) vertebral chondrification and (6) vertebral ossification. **b** : Ossification of subaxial cervical, thoracic and lumbar vertebrae: This follows chondrification and typically occurs after 9 weeks of gestation. One dorsal and one ventral primary ossification centre forms for each vertebral body; these unite to create a centrum, which develops into three primary loci at the conclusion of embryonic development. One of these loci eventually develops into the vertebral body while the other two develop into one half of the eventual vertebral arch. **c**: Development of C1 (atlas): The atlas develops from three centres of ossification separated by cartilaginous synchondroses—one for body and two for lateral masses. The sequence of appearance and fusion of the ossification centres as noted in the illustration. **d**: Development of the C2 (axis): Six centres of ossification separated by four cartilaginous synchondroses. Sequence of appearance and fusion of the ossification centres as noted in the illustration

Development of the spine and spinal cord occurs side by side, intimately intertwined. The vertebral column develops in distinct phases, as illustrated below (Fig. 1a) [5]. Spinal cord development occurs in four basic embryological stages including ***gastrulation*** (weeks 2 to 3), ***primary neurulation*** (weeks 3 to 4) and ***secondary neurulation*** (weeks 5 to 6). Simply put, ***gastrulation*** is the process by which a bilaminar embryonic disk is converted into a trilaminar structure by migration of epiblastic cells through the Hensen’s node (knoblike termination of the primitive streak containing totipotential cells) to the epiblast/hypoblast interface—a process that ultimately results in formation of the intervening mesoderm/notochord. The notochord plays an important role in somitic differentiation, vertebral chondrogenesis and segmentation [6].

Since the notochord is created during gastrulation, multisystem abnormalities typically coexist with notochordal developmental anomalies [7]. After the notochord interacts with the overlying ectoderm to form the neuroectoderm, neural tube closure occurs to complete the process of primary neurulation. The sacrum and coccyx develop last at about 31 days of gestation [5] from a large aggregate of undifferentiated cells (caudal cell mass) representing remnants of the primitive streak. Underlying embryological processes include canalisation (secondary neurulation) and retrogressive differentiation.

The craniovertebral junction (CVJ) is embryologically unique and complex. Four occipital sclerotomes contribute to formation of the occipital bone, clivus and occipital condyles, anterior arch of the atlas and the apical, cruciate and alar ligaments. The posterior arch of the atlas is derived from both the first occipital and first cervical sclerotomes. The axis is derived from both the fourth occipital and first and second cervical sclerotomes. The ventral portion of the first cervical sclerotome forms most of the odontoid process [3, 5].

The final steps in vertebral formation, chondrification and ossification occur after 6 and 9 weeks respectively. Chondrification of the CVJ begins at 45 days and chondrification of the C1 anterior arch begins at 50 to 55 days of gestation [5, 8].

**Fig. 2:**
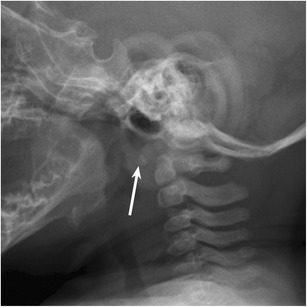
Normal lateral radiograph of the cervical spine in a 13 month old reveals the ossification centre of the body of the atlas. The posterior arches have not yet appeared

The subaxial cervical, thoracic and lumbar vertebral bodies each have two ossification centres, which merge, and single ossification centres on each side of the vertebral arch (Fig. 1b). Ossification centres of the first and second cervical vertebrae follow a predictable sequence and timing of appearance and fusion [9, 10] (Figs. 1c,d and 2). The atlas should be completely visible on cervical spine radiographs by 6–7 years of age [10]. The body of C2 usually fuses to the neural arches and dens between 3 and 6 years of age, and the dens fuses to the ossiculum terminale at 12 years [10].

At the sacrococcygeal levels, the first three sacral elements contain an additional pair of ossification centres. Fusion of sacral vertebrae begins early in puberty and is complete in the middle part of the 3rd decade. Ossification of the first coccygeal segment begins between 1 to 4 years of age; remaining coccygeal segments ossify craniocaudally between 5 to 20 years of age [11].

Vertebral malformations have been classified based on the underlying embryopathy [5, 12] (ESM_1). Developmental variances leading to transitional vertebrae at the thoracolumbar and lumbosacral junctions and developmentally short/absent pedicles have been identified as a separate category. The subsequent paragraphs outline the embryology, clinical and radiological manifestations of commonly encountered vertebral malformations (ESM_1).

### Abnormal gastrulation

The embryological processes underlying this spectrum of malformations involve disorders resulting from abnormal development of the notochord during gastrulation. These typically manifest with malformation of the neuraxis and axial skeleton involving tissues derived from *all* three primary germ cell layers [13]. Broadly, these include disorders of midline integration of the notochord (split notochord syndrome, split cord) and disorders of notochordal formation (caudal agenesis and segmental spinal dysgenesis) (ESM_2).

#### Split notochord syndrome

This results from splitting of the notochord leading to a persistent connection between the ventral endoderm and the dorsal ectoderm. The most severe manifestation is a ***dorsal enteric fistula*** through which the intestinal cavity communicates with a dorsal skin surface to the midline, traversing all the intervening structures [11]. Variants on this theme include a dorsal bowel hernia and diverticuli, duplications, cysts or sinuses along this anomalous tract [11].

#### Split cord/diastematomyelia (Fig. 3a,b)

This refers to sagittal division of the spinal cord into two symmetric/asymmetric hemicords, each containing a central canal, one dorsal and one ventral horn and each invested by its own leptomeninges [11].

**Fig. 3:**
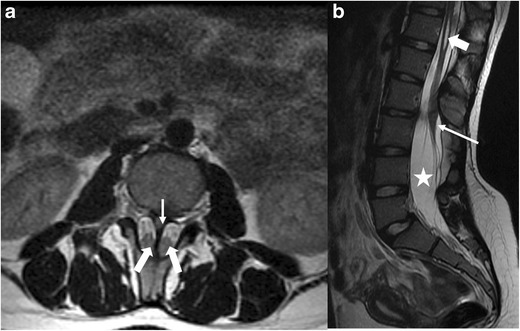
**a**-**b**: Split cord/diastematomyelia in 15 year old: Axial (*a*) and sagittal T2-weighted (*b*) MR images of the lumbar spine reveals an osseous septum (*thin arrow* on *a*) dividing the spinal cord into symmetric halves (*thick, block arrows* on *a* ). Notice that the cord is low-lying and posteriorly positioned within the spinal canal (*thin arrow* on *b*) and contains a syrinx (*block arrow* on *b* ). Associated dural ectasia (*star* on *b*) is also seen

Erroneous specification of the rostrocaudal positional encoding of prospective notochordal cells can result in inadvertent apoptosis of notochordal precursors and subsequent interference with primary and secondary neurulation, leading to disordered formation of the notochord; these can manifest as ***caudal/sacral agenesis*** or ***segmental spinal dysgenesis***.

#### Caudal/sacral agenesis (Fig. 4a-e)

This represents a group of rare structural vertebral anomalies affecting the formation of the distal spinal segments and the spinal cord [14]. These abnormalities are characterised by varying degrees of developmental failure involving the lower extremities, sacrococcygeal and occasionally distal thoracolumbar vertebrae and the corresponding spinal cord segments [15]. Based on the underlying embryopathy, there are two broad variants. ***Type I*** involve disorders of both primary and secondary neurulation, thereby resulting in pronounced agenesis of the distal vertebrae with coexistent aplasia of the caudal metameres of the spinal cord, whereas ***type II*** involve disorders of only secondary neurulation, resulting in less severe vertebral dysgenesis, absence of only the tip of the conus medullaris and association with tethered cord syndrome [16]. Associated myelomeningoceles, diastematomyelia, tethered spinal cord, thickened filum terminale and lipomas of the cord may be present.

**Fig. 4:**
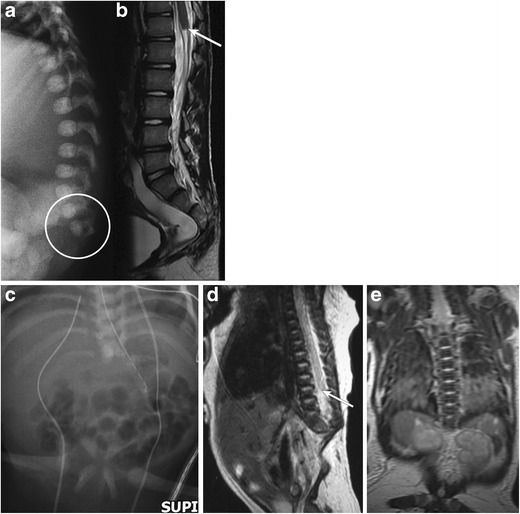
**a**-**b**: Caudal/sacral agenesis: Lateral radiograph of the spine (*a*) in one infant reveals absence of the distal scacrococcygeal segments. Accompanying sagittal T2-weighted MR image (*b*) of the lumbar spine reveals abnormal truncation of the conus medullaris (*arrow*). **c**-**e**: Caudal agenesis (another neonate): Frontal radiograph of the abdomen (*c*) in a neonate reveals absence of the entire lumbar and sacrococcygeal spine; the iliac bones are dysmorphic and abnormally close, though separate. Accompanying sagittal (*d*) and coronal (*e*) MR images of the spine reveal absence of the lumbar-sacrococcygeal segments of the spine. Abnormal high termination and truncation of the spinal cord is noted (*arrow* on *d*)

**Fig. 5:**
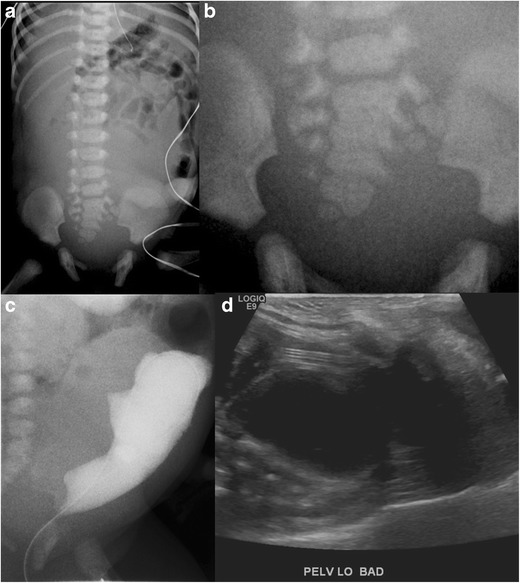
**a**-**d**: Dysmorphic sacrum with imperforate anus and presacral cystic mass: Frontal abdominal radiograph of a newborn demonstrates absence of bowel gas in the pelvis (*a*) with a history of no passage of meconium; the patient had ano-rectal atresia. Pelvic radiograph (*b*) demonstrates an abnormal sacrum. Retrograde cystogram (*c*) demonstrates an elongated urinary bladder with extrinsic indentations along its posterior aspect, suggesting a pelvic mass. Pelvic ultrasound (*d*) demonstrates a lobulated cystic pelvic mass; pathology was consistent with teratoma

Caudal agenesis may be a component in syndromic complexes including OEIS (concurrent omphalocele, cloacal extrophy, imperforate anus and spinal deformities) [17], Currarino’s triad [sacral dysgenesis, a pre-sacral mass and anorectal malformation] (Fig. 5a-d) and VACTERL anomalies (vertebral anomalies, imperforate anus, cardiac malformations, tracheoesophageal fistula, renal anomalies and limb deformities) [7].

**Fig. 6:**
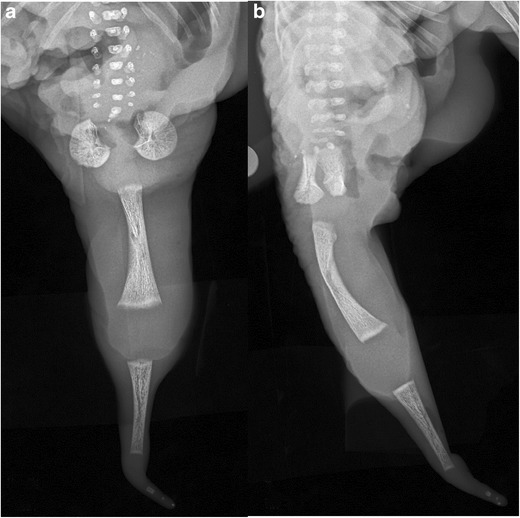
**a**-**b**: Sirenomelia (“mermaid sequence”): Post-mortem AP (*a*) and lateral (*b*) radiographs of the lower body of a prematurely terminated foetus demonstrates absent sacrum and fused bilateral lower extremities. This anomaly is incompatible with life

A discussion of sacral dysgenesis is incomplete without a mention of ***sirenomelia/mermaid sequence*** (Fig. 6a-b). This rare, lethal congenital anomaly is characterised by an absent sacrum, rectum and bladder, fused lower extremities and bilateral renal agenesis [18]. The underlying aetiology is believed to be related to vascular disruption to the caudal portion of the embryo [18].

#### Segmental spinal dysgenesis (Fig. 7a-e)

This is a complex spinal dysraphism. Thoracic, lumbosacral segments of the spine may be affected. The clinical/radiological definition includes: (1) segmental agenesis or dysgenesis of the lumbar or thoracolumbar spine; (2) segmental abnormality of the underlying spinal cord and nerve roots; (3) congenital paraplegia or paraparesis; (4) congenital lower limb deformities [16]. The most extreme cases manifest with absence of the spinal cord at the affected level and focal aplasia of the vertebral body, resulting in acute kyphosis [7]. The lower spinal cord is thick and abnormally low [7]. Associated lipomas, dermal sinuses or hydromyelia may be present. Horseshoe kidney is commonly associated [7].

**Fig. 7:**
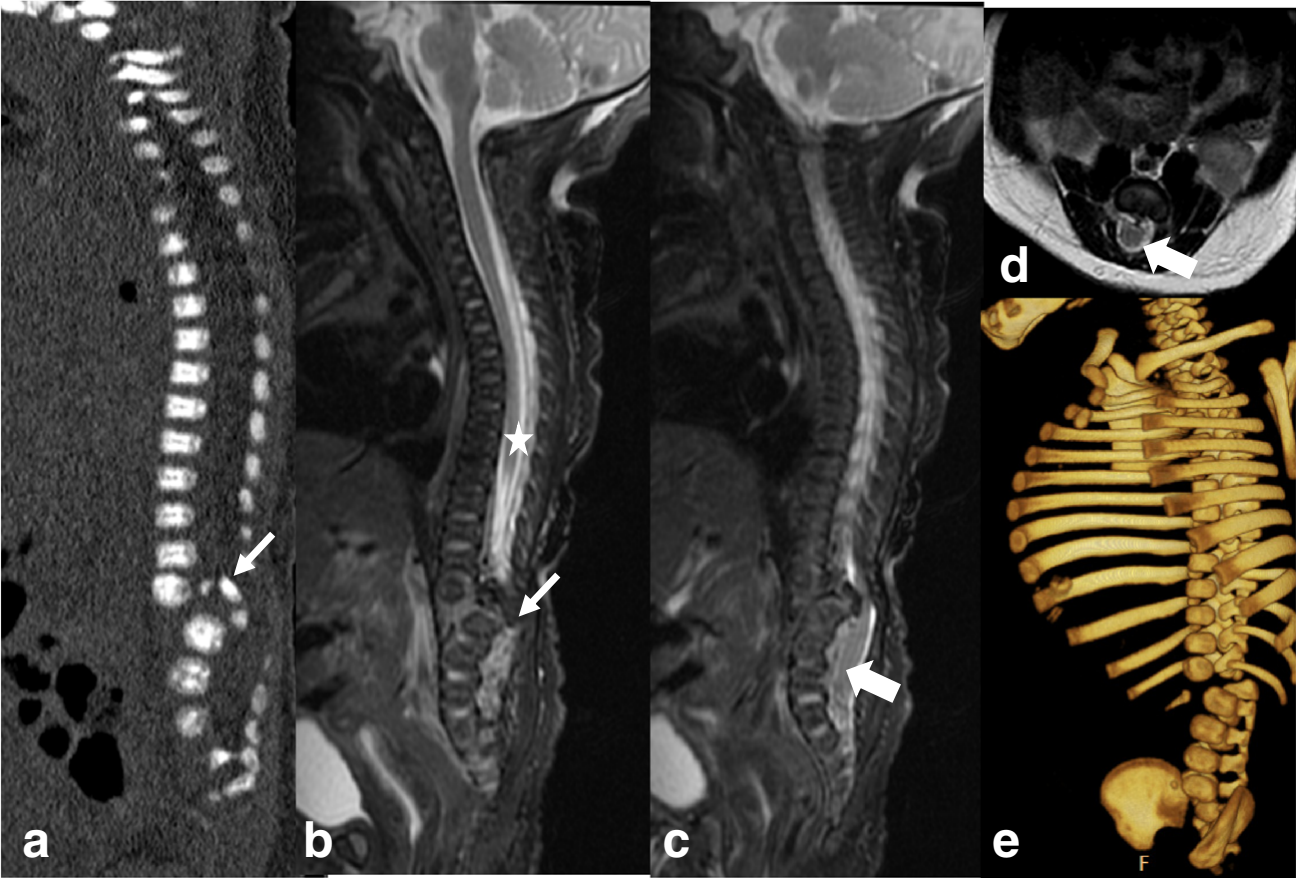
**a**-**d**: Segmental spinal dysgenesis in a neonate: Sagittal CT reconstruction (*a*) and sagittal T2 MR (*b*) images of the spine in the neonate with congenital paraplegia show absence of L1 and posterior dislocation of a hypoplastic L2 vertebra into the spinal canal (*arrows* on *a* and *b*). The spinal cord (*star* on *b*) is absent over the affected segments of the spine, but is thick and abnormally low further distally (*block arrows* on *c* and *d*). The 3D bony reformats from concomitantly performed CT (*e*) redemonstrate absence of L1 and hypoplasia of the posteriorly dislocated L2

### Abnormal alignment of sclerotomal rests

#### Hemivertebrae

These result from complete failure of formation of one of the two paired cartilaginous centres of the developing vertebra, secondary to tardy development of a somite on one side resulting in a caudal metameric segmental shift of one somatic column relative to another, leading to an unpaired sclerotome and resultant hemivertebra [5]. The contralateral vertebral centrum and corresponding dorsal vertebral arch are characteristically absent. The ipsilateral posterior arch, though present, is incorporated into the vertebral arch above or below.

Hemivertebrae can occur sporadically or in association with spinal dysraphisms, skeletal, cardiac, genitourinary and gastrointestinal anomalies; the latter category of infants is predisposed to increased perinatal mortality [19].

**Fig. 8:**
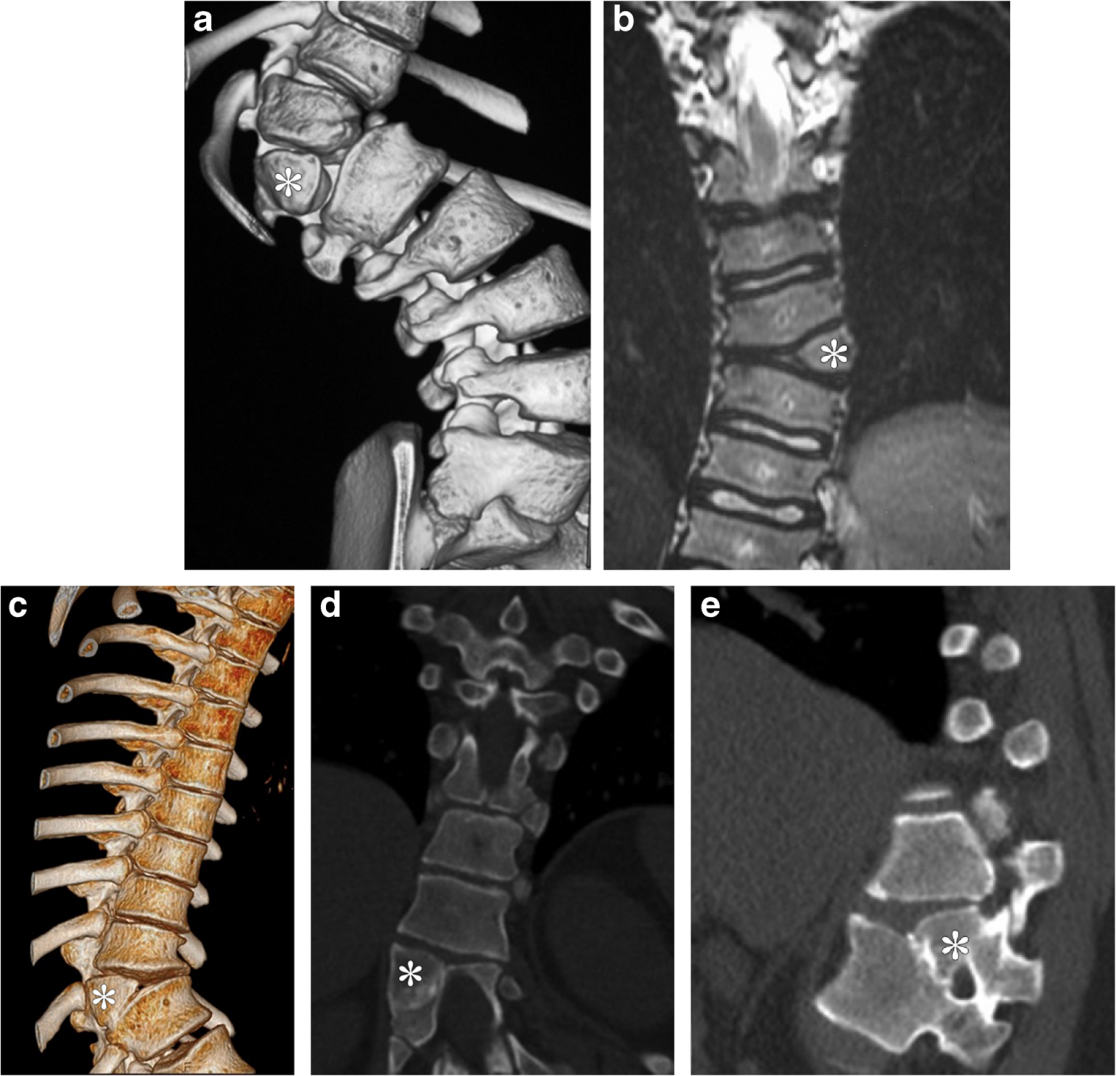
**a**-**b**: Segmented hemivertebra in two patients: The 3D reconstruction from a non-contrast CT of the spine (*a*) and coronal T1-weighted MR image of different levels of the thoracic spine (*b*) demonstrate a laterally located wedge of bone composed of half a vertebral body (marked by *asterisks*). Note that there is associated scoliotic deformity, more pronounced on *a*. This variety of hemivertebra, being clearly separate from its neighbour above and below, has the most potential for impacting spinal curvature. **c**-**e**: Semisegmented hemivertebra in 16 year old: 3D reconstruction (*c*) and coronal and oblique sagittal reconstructions from a non-contrast CT of the spine (*d* and *e*) demonstrate a laterally located wedge of bone composed of half a vertebral body (marked by asterisks). Note that this variant is attached to its neighbouring inferior vertebra and has only the superior disc space adjacent to it

Based on their growth pattern, hemivertebrae can be classified into the **fully segmented** (nonincarcerated and incarcerated variants) (Fig. 8a-b), **semisegmented** (Fig. 8c-e) and **nonsegmented**. The **fully segmented, nonincarcerated** hemivertebra has a normal disk space above and below and causes the most disruption of normal spinal curvature; the **incarcerated** variant is set into defects in the vertebra above and below and leads to less pronounced disruption of spinal curvature [20]. A **semisegmented** hemivertebra only has one disk space adjacent to it. A **nonsegmented** hemivertebra lacks disk spaces on either side, being attached to both its neighbours; this variant is least likely to result in scoliosis [19].

### Disordered vertebral formation from sclerotomal precursors

Embryologically, these occur as a consequence of disruption of the somitic mesoderm during gastrulation, somites during segmentation or sclerotomal precursors during the membranous phase. Examples include wedge vertebrae and less than 10% of hemivertebrae.

#### Wedge vertebrae

**Fig. 9:**
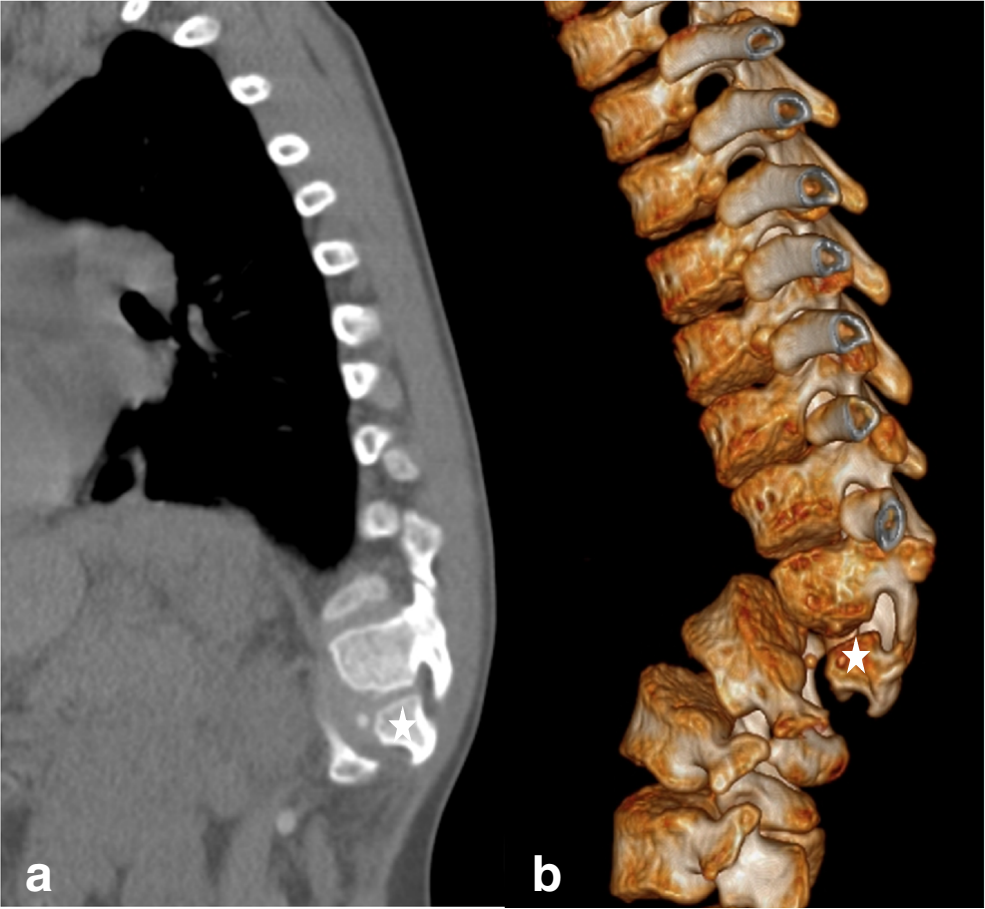
**a**-**b**: Wedge vertebra at T12: Sagittal CT (*a*) and volumetric CT reconstruction of the spine (*b*) demonstrate a small, wedge-shaped T12 (*stars* on *a* and *b*) with associated focal kyphosis; there was associated scoliosis (not depicted)

These arise as a result of diminished cell contribution from the ventral sclerotome and thereby diminished centrum height (Fig. 9a-b). As a more severe manifestation of this spectrum, the posterior vertebral arches may be deficient and hypoplastic posterior segments of the adjacent vertebrae may fuse to form a ***dorsolateral unsegmented bar*** with associated rib anomalies at the thoracic location. In yet more severe forms, irregular ***hemivertebrae*** may result. These are distinct from hemivertebrae arising secondary to hemimetameric shifts, cross the midline to a variable degree and comprise less than 10% of all hemivertebrae [5].

### Defective vertebral segmentation

These abnormalities occur as a result of failed segmentation of somites. These include ***block vertebrae, unsegmented bars*** and ***congenital cervical spine fusions*** as seen with ***Klippel-Feil syndrome*** [21].

#### Block vertebrae

**Fig. 10:**
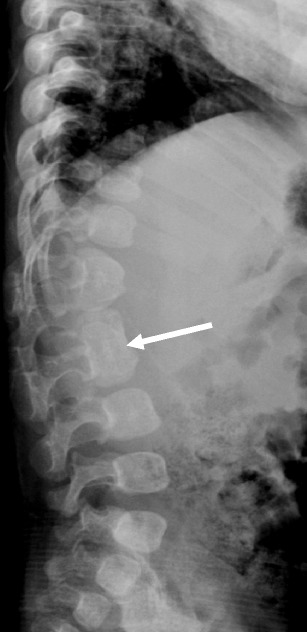
Ventral block vertebra in 8 month old: Lateral radiograph of the spine demonstrates fused L2 and L3 vertebral bodies (*arrow*). The T12 and L1 vertebral bodies were also partially fused

are congenitally fused vertebrae and may be ventral (affecting only the vertebral body) (Fig. 10), dorsal (affecting the vertebral arch) or both. Unilateral *unsegmented* ***bars*** represent a common cause of congenital scoliosis. These lack growth plates but the unaffected side of the spine continues to grow leading to spinal deformities [20]. These may coexist with hemivertebrae. Spinal curvature deteriorates at an average rate of 5° or greater annually [20].

#### Klippel Feil syndrome

**Fig. 11:**
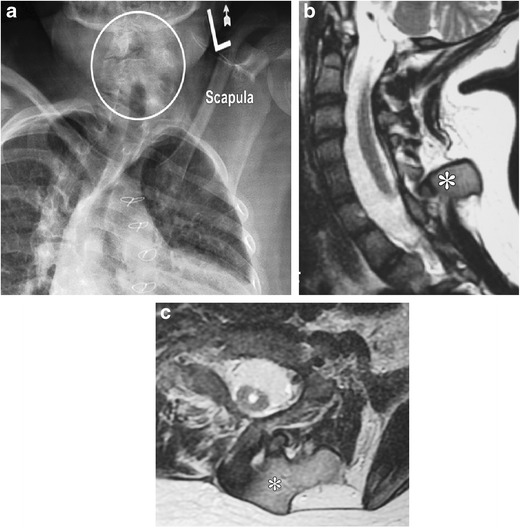
**a**-**c**: Klippel-Feil abnormality with Sprengel shoulder: Frontal radiograph of the neck and upper chest in an 11 year old demonstrates marked elevation of the left scapula. Also seen are segmentation anomalies of the cervical spine (demarcated by *circle* on *a*). Sagittal (*b*) and axial (*c*) T2-weighted MR images demonstrate a large bony omovertebral bar (marked by *asterisks* on both images) extending between the medial border of elevated scapula and spinous process of a lower cervical vertebra

This represents congenital partial or complete fusion of two or more adjacent cervical vertebrae [22] (Fig. 11a-c). The classic triad including short neck, limited neck motion and low posterior hairline is present in 50% of these patients [23]. According to one retrospective review, the most common lesion is an isolated fusion of C2 and C3 [24]. Intraspinal abnormalities such as Arnold-Chiari malformation type I, syringomyelia, diastematomyelia or tethered cord may be associated. These abnormalities increase the vulnerability of the cord to trauma with even minor injury [22, 25, 26]. Reduced neck mobility in these patients makes emergency endotracheal intubation challenging [22].

On radiographs, vertebral bodies typically have narrow, tall configurations and decreased anteroposterior dimensions with absent or small intervening disks. Associated fusion of posterior elements, occipitalisation of the atlas, congenital scoliosis, kyphosis and ***Sprengel deformity*** (see below) may be present. Flexion and extension radiographs should be routinely performed in these patients to assess for potential instability [27]. Patients should be counselled to protect themselves against injury.

#### Sprengel deformity

This refers to a dysmorphic, high-positioned scapula at birth that results from lack of normal caudal migration of the scapula during embryogenesis. The scapula often has a convex medial margin, concave lateral margin, decreased height to width ratio and associated hypoplasia of the scapular muscle [28]. CT can identify associated congenital scoliosis and omovertebral bars and help in surgical planning [28].

### Disordered vertebral alignment

This entity results from simple mechanical buckling of the embryo between the 4th and 6th embryonic weeks, after neurulation but before chondrification [29]. This commonly occurs at or near the thoracolumbar junction and manifests as ***congenital vertebral dislocation***. The spinal canal at the affected level is typically widened, pedicles of the more cephalad vertebra are elongated, and the dorsal vertebral arches are dysraphic. The spinal cord is frequently low-lying, although intact across the lesion. Usually, patients are neurologically intact or present with subtle neurological deficits. Associated tracheoesophageal fistula and unilateral renal agenesis may be present.

### Disordered fusion of the sclerotome, chondrification or ossification centres

Vertebral malformations attributed to disordered assimilation or fusion include ***butterfly vertebra*** **and** ***dysplastic spondylolysis*** [30].

#### Butterfly vertebra (Fig. 12)

These manifest as a sagittal cleft between the unfused sclerotomal pairs. Moulding of adjacent vertebral bodies towards the midsagittal constriction may or may not be present. Although usually asymptomatic, this can be mistaken for compression, burst or wedge fractures on imaging [30].

**Fig. 12:**
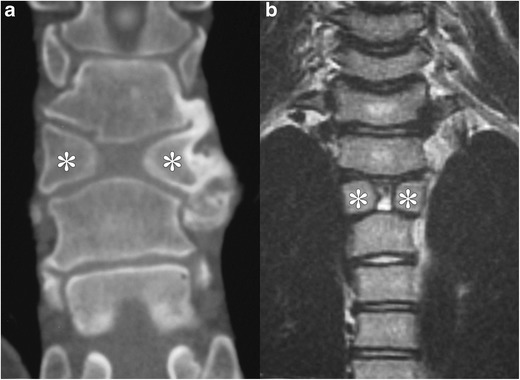
Butterfly vertebra in two patients: Coronal reformatted image from a non-contrast CT of the spine (*a*) and coronal T1-weighted MR image of the upper thoracic spine (*b*) both demonstrate separate ossification centres on each side of the vertebral body (marked by *asterisks*). Note that there is moulding of the adjacent vertebral bodies towards the midsagittal constriction, especially pronounced on a

Localised failure of fusion of ventral and dorsal ossification centres can result in malformed vertebral pedicles or facets (***dysplastic spondylolysis***). When this process occurs dorsally, ***spina bifida occulta*** can result.

### Disordered ossification

Failure of development of the vertebral centrum during late stages of gestation manifests as isolated ***hypoplasia or aplasia of the vertebral centrum***, without corresponding alterations in the dorsal vertebral arch. On imaging, part or all of the vertebral centrum may be absent with intact pedicles and posterior body up to the neurocentral synchondrosis. Also included in the spectrum are ***dorsal hemivertebra*** with isolated absence or wedging of the ventral portion of the centrum [5].

### Craniovertebral junction (CVJ) malformations

**Fig. 13:**
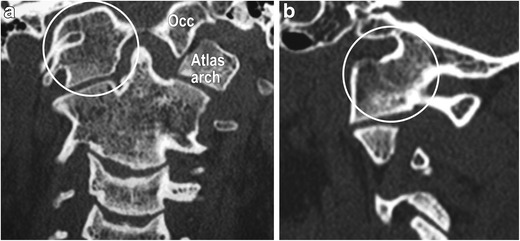
Atlanto-occipital assimilation in a 19-year-old female: Coronal (*a*) and sagittal (*b*) reformats from a cervical spine CT reveal abnormal fusion of the right-sided arch of atlas with the corresponding occipital condyle (*arrows*). The structures are clearly separate on the left

**Fig. 14:**
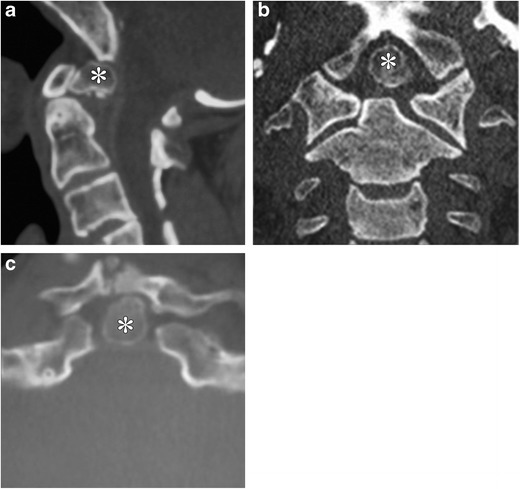
Dystopic os odontoideum in a 19 year old: Sagittal (*a*), coronal (*b*) and axial (*c*) images from a non-contrast CT in a 19 year old demonstrate a well-corticated ossific density along the superior margin of the dens (marked by *asterisks* on images *a*, *b* and *c*). The dens itself is relatively small with a foreshortened base (*block arrow* on *b*). Note that the os odontoideum is located somewhat posterior to the C2 body and is therefore not in its expected anatomic location

The unique embryology of the craniovertebral junction predisposes this region to some unique developmental errors (ESM_3). Abnormalities of the CVJ can impact the cervical spinal cord, brainstem, cerebellum, cervical nerve roots, lower cranial nerves and vascular supply to these structures. ***Atlanto-occipital assimilation*** is the most common congenital anomaly of the craniovertebral junction (Fig. 13a-b). *Os odontoideum* (Fig. 14a-c) refers to a circumferentially corticated ossific fragment separated from a hypoplastic or foreshortened base of the dens [31, 32]. The two subtypes include **orthotopic** and **dystopic** [31]. This can be ***stable*** or ***unstable*** [31]; the latter, if symptomatic, should be surgically addressed [33]. Klippel-Feil anomaly, spondyloepiphyseal dysplasia, Down syndrome and Morquio syndrome may be associated.

#### Persistent ossiculum terminale (Fig. 15a-b)

This condition exists when the ossiculum terminale (secondary ossification centre located at the superior margin of dens) fails to fuse with the dens, a process that normally occurs early in the 2nd decade of life. Usually incidental, this may mimic a type I dens fracture; both are typically stable [31]. Smooth, corticated margins characterise a persistent ossiculum terminale, whereas irregular, lucent margins are seen with acute type I fractures of the dens, enabling differentiation [31].

**Fig. 15:**
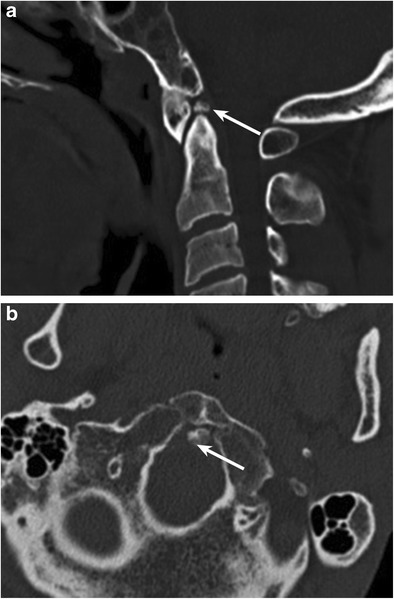
Persistent ossiculum terminale: Sagittal (*a*) and axial (*b*) images from a non-contrast CT of the cervical spine in a 32 year old demonstrate a small, well-corticated ossific density along the superior margin of the dens (*arrows* on images *a* and *b*). This likely represents the persistent ossiculum terminale, which arises as a result of failure of fusion of the secondary ossification centre with the superior aspect of the dens. This entity can be mistaken for a remote type I fracture of the dens

**Fig. 16:**
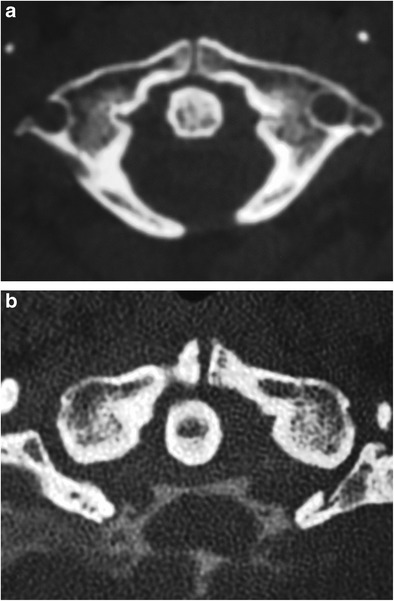
Hypoplasia/aplasia of the posterior arch of C1. Axial CT images at the level of the atlas on two patients reveal cervical vertebral cleft (posterior rachischisis) in *a* and absent posterior arches in *b*. Also note anterior vertebral body clefts on both images

Other abnormalities of this region include congenitally absent anterior/posterior atlas arches (Fig. 16a-b), odontoid tip/entire odontoid, bifid odontoid, fused odontoid tip with inferior clivus, with basilar invagination and hypoplastic occipital condyles [3] (ESM_3).

### Developmental variance

This category includes vertebral malformations that do not strictly fall under any of the above categories but arise secondary to variances in development. Entities discussed under this category include transitional vertebrae [34] and congenitally short/absent vertebral pedicles.

#### Thoracolumbar and lumbosacral junctional variances

***Thoracolumbar junctional variances*** manifest either as an increase or decrease in the number of rib-bearing thoracic vertebrae or altered appearances of the costal processes of the most caudal thoracic or the uppermost lumbar vertebra. ***Lumbosacral transitional vertebrae*** have been described as either sacralisation of the lowest lumbar segment or lumbarisation of the most superior sacral segment of the spine [35]. On imaging, these are best seen with CT. Clinically, these can be associated with back pain/“Bertolotti syndrome” [36] and can lead to nomenclature and/or surgical errors [37]. Castellvi et al. proposed a radiographic classification system based on the morphological characteristics of these vertebrae [38]. Treatment may be conservative or surgical.

#### Congenital/developmental spinal stenosis secondary to short pedicles (Fig. 17a–b)

This developmental variant manifests as narrowing of the anteroposterior dimension of the spinal canal to less than 10 mm or thecal sac cross-sectional area less than 77 ± 13 mm² [39]. This occurs most commonly at the level of the lumbar spine, where it may present with neurogenic intermittent claudication and radiculopathy [40]. The spinal cord is vulnerable to trauma or disk herniation. In one prospective cohort study [39], the measured pedicle lengths among congenitally stenotic patients approximated 6 mm whereas the control group had pedicle lengths closer to 9 mm.

**Fig. 17:**
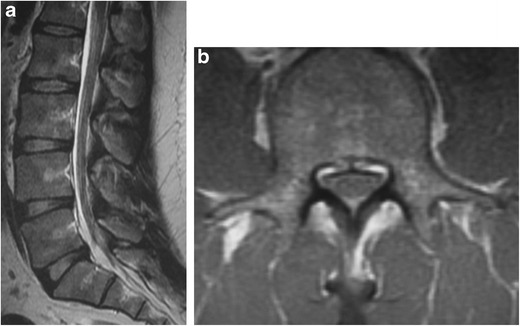
Congenital stenosis of the spinal canal: Sagittal (*a*) and axial (*b*) T1-weighted MR images of the lumbar spine reveal narrowed anteroposterior diameter of the spinal canal (< 10 mm)

#### Pedicular agenesis [41]

This unusual disorder manifests with isolated agenesis of a vertebral pedicle, most commonly C5 or C6. Imaging findings include misleading appearance of enlarged ipsilateral neural foramen, dysplastic dorsally displaced ipsilateral lamina and a dysplastic ipsilateral transverse process. Although a stable congenital anomaly, this can be mistaken for acute trauma [42].

## Conclusion

Structural abnormalities of the spine can occur at multiple levels and have a variety of imaging and clinical manifestations. The radiologist plays an important role in assessing these abnormalities and can alert the clinician to the likelihood of a serious complication arising secondary to such abnormalities and assist in the pre-operative workup and postoperative follow-up.

## Electronic supplementary material

ESM 1 (DOCX 20.4 kb)

ESM 2 (DOCX 17.9 kb)

ESM 3 (DOCX 17.3 kb)
