# Supplementary material for: Malformed vertebrae: a clinical and imaging review
Source: Insights Imaging. 2018 Apr 3;9(3):343–55. doi: 10.1007/s13244-018-0598-1 (PMC5991006; doi:10.1007/s13244-018-0598-1)
Supplement: Supplementary file 1 — (DOCX 20.4 kb) [file 13244_2018_598_MOESM1_ESM.docx]

| **Abnormal gastrulation** | **Abnormal alignment of sclerotomal rests** | **Disordered vertebral formation from sclerotomal precursors** | **Defective vertebral segmentation** | **Disordered vertebral alignment** | **Disordered fusion of sclerotome, chondrification or ossification centers** | **Disordered ossification** | **Craniovertebral junction malformations** | **Developmental variance** |
| --- | --- | --- | --- | --- | --- | --- | --- | --- |
| Errors of notochord integration  -Split notochord  -Split cord  Errors of notochord formation  -Syndrome of caudal regression  -Segmental spinal dysgenesis | Hemivertebrae | Wedge vertebrae  Dorsolateral unsegmented bar  Hemivertebrae | Klippel-Feil syndrome  Block vertebrae  Tripediculate vertebrae | Congenital vertebral dislocation | Butterfly vertebra  Dysplastic spondylolysis  Spina bifida occulta | Hypoplasia/  aplasia of vertebral centrum  Dorsal hemivertebra | Atlanto-occipital assimilation  Os odontoideum  Ossiculum terminale  Congenital absence of C1 anterior/posterior arches/odontoid tip/entire odontoid  Basilar invagination  Occipital condyle hypoplasia  Fusion/segmentation defects at the craniovertebral junction | Transitional vertebrae (thoracolumbar, lumbosacral)  Developmental spinal stenosis  Pedicular agenesis |
