# Supplementary material for: Malformed vertebrae: a clinical and imaging review
Source: Insights Imaging. 2018 Apr 3;9(3):343–55. doi: 10.1007/s13244-018-0598-1 (PMC5991006; doi:10.1007/s13244-018-0598-1)
Supplement: Supplementary file 2 — (DOCX 17.9 kb) [file 13244_2018_598_MOESM2_ESM.docx]

| **Entity** | **Underlying embryopathy** | **Clinical manifestations** | **Radiological findings** |
| --- | --- | --- | --- |
| Split notochord | Errors of midline integration of the notochord | ***Dorsal enteric fistula:*** Exposed mucous membrane/bowel ostium in the dorsal midline passing meconium  ***Intraspinal enteric cysts:*** Intermittent/progressive radicular pain, ultimately progressing to myelopathy | ***Dorsal enteric fistula:*** Vertebral body abnormalities; extension of intra-abdominal structures through vertebral body defects  ***Intraspinal enteric cysts:*** Widened spinal canal at the affected site  Intradural, extramedullary cystic structures ventral or ventrolateral to the spinal cord  Hemivertebrae, partial fusion |
| Split cord |  | Skin stigmata (hairy patch, nevi, lipomas, dimples and hemangiomas)  Neuro-orthopedic syndrome (weakness/muscle wasting in one leg with ipsilateral clubfoot) | Bony/cartilaginous spur dividing the spinal cord; usually lumbar, less commonly thoracic and rarely cervical  Low-lying conus medullaris/ thickened filum terminale  Myelocele/meningomyelocele of the undivided spinal cord  Thickened vertebral laminae, sometimes fused with the contralateral laminae of the adjacent level *(intersegmental laminar fusion)*  Spina bifida  Hemivertebrae, butterfly vertebrae, block vertebrae |
| Sacral dysgenesis/Caudal regression | Disorder of formation involving a segment of the notochord | Minor distal muscle weakness involving the feet to complete sensorimotor paralysis involving both lower extremities  Motor deficits>> sensory deficits  Neurogenic urinary bladder | Partial/complete agenesis of sacrum/coccyx with or without involvement of the thoracic lumbar spine; fused iliac wings  Thecal sac and spinal canal narrowing inferior to the lowest normal vertebral level  Abrupt, high termination of the cord seen with Type I abnormalities  Absence of only tip of the conus medullaris and association with tethered cord syndrome seen with Type II abnormalities |
| Segmental spinal dysgenesis |  | Marked focal kyphosis  Lower extremity abnormalities: Equinovarus abnormalities of the feet, lower extremity hyperreflexia, flexion contractures of the hips and knees  Bladder dysfunction | Focal kyphotic deformity with hypoplastic or absent single or multiple vertebral bodies on plain films    Marked narrowing or complete focal absence of the spinal cord at the affected level  Abrupt, sometimes blunted terminus of the cord above the affected segment  Lower spinal cord thick and abnormally low |
