# Supplementary material for: Malformed vertebrae: a clinical and imaging review
Source: Insights Imaging. 2018 Apr 3;9(3):343–55. doi: 10.1007/s13244-018-0598-1 (PMC5991006; doi:10.1007/s13244-018-0598-1)
Supplement: Supplementary file 3 — (DOCX 17.3 kb) [file 13244_2018_598_MOESM3_ESM.docx]

| **Entity** | **Underlying embryopathy/abnormality** | **Clinical manifestations** | **Radiologic manifestations** |
| --- | --- | --- | --- |
| Atlanto occipital assimilation | Failed segmentation between the fourth occipital sclerotome and the first cervical sclerotome | C1/C2 instability  Torticollis (if unilateral) | Abnormal fusion of the occipital condyles with the anterior-posterior arches or lateral masses of C1 or combinations of the above; can be complete, partial, unilateral and bony or fibrous  Basilar invagination |
| Os odontoideum | Failure of fusion between odontoid tip (fourth cervical sclerotome) and the odontoid process (first cervical sclerotome) | May be asymptomatic  If unstable, central canal stenosis at the craniocervical junction with associated cord compression can occur | Circumferentially corticated ossific fragment separate from the hypoplastic or foreshortened base of the dens; hypertrophied anterior arch of C1 with incompetence/instability of cruciate ligament (zone of high spinal cord signal seen on T2-weighted MR imaging)  ***Orthotopic*** variant located along the superior margin of the base of the dens; when unstable, articulates with the anterior arch of C1 independent of the base of the dens  ***Dystopic*** variant is not in the expected anatomic location and can be fused to the basion/inferior margin of the clivus |
|  |  |  |  |
| Persistent ossiculum terminale | Failure of fusion of secondary ossification center located at the superior margin of the dens to the dens | Usually an incidental, asymptomatic finding (mimics a type I dens fracture) | Corticated ossific fragment adjacent to the superior margin of the dens  V-shaped cartilaginous cleft at the superior aspect of the dens |
| Congenital absence of C1 anterior arch/posterior arch/odontoid tip/entire odontoid | Inadequate development of the corresponding sclerotome; incomplete chondrification/ossification | Usually incidental, occasionally pain/deformity, myelopathy | Absence of the corresponding portion of the atlas  Posterior arch defects much more common than anterior arch defects |
| Basilar invagination | Insufficient paraxial mesoderm | Short, webbed neck, painful and limited neck mobility  Can be associated with Chiari-1 malformation | Odontoid process protrudes through the foramen magnum (inadequate visualization of C1/2 facet complex on an open mouth radiographic projection)  Impingement of spinal cord/nerve roots seen by MR |
